# Supplementary figures and images for: Strategies to prevent hospital readmission and death in patients with chronic heart failure, chronic obstructive pulmonary disease, and chronic kidney disease: A systematic review and meta-analysis
Source: PLoS One. 2021 Apr 22;16(4):e0249542. doi: 10.1371/journal.pone.0249542 (PMC8062060; doi:10.1371/journal.pone.0249542)

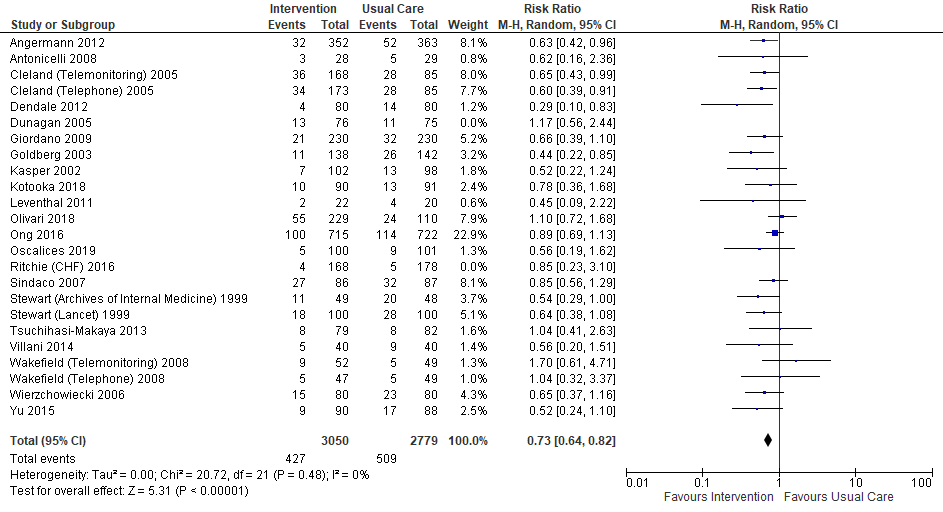

Supplement: S1 Fig — (DOCX) [file pone.0249542.s002.docx]

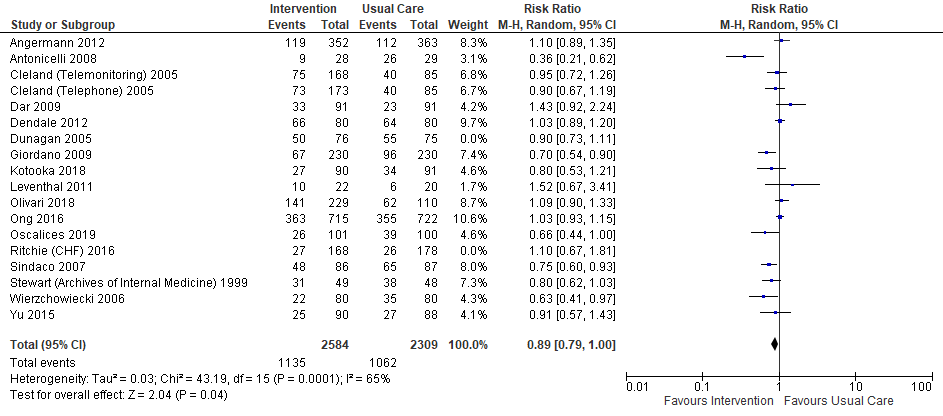

Supplement: S2 Fig — (DOCX) [file pone.0249542.s003.docx]

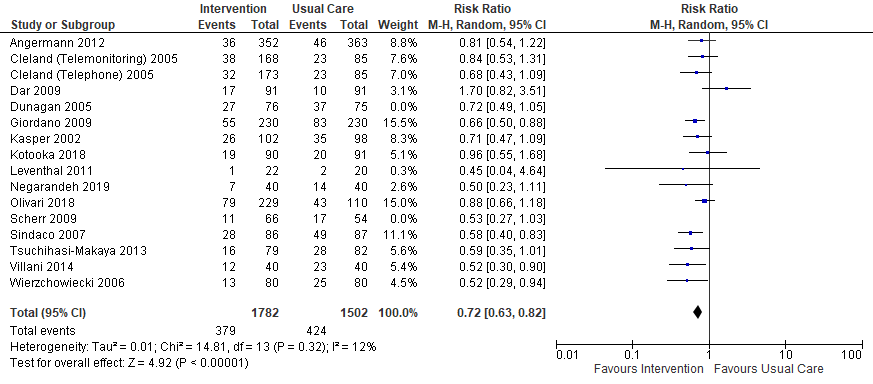

Supplement: S3 Fig — (DOCX) [file pone.0249542.s004.docx]

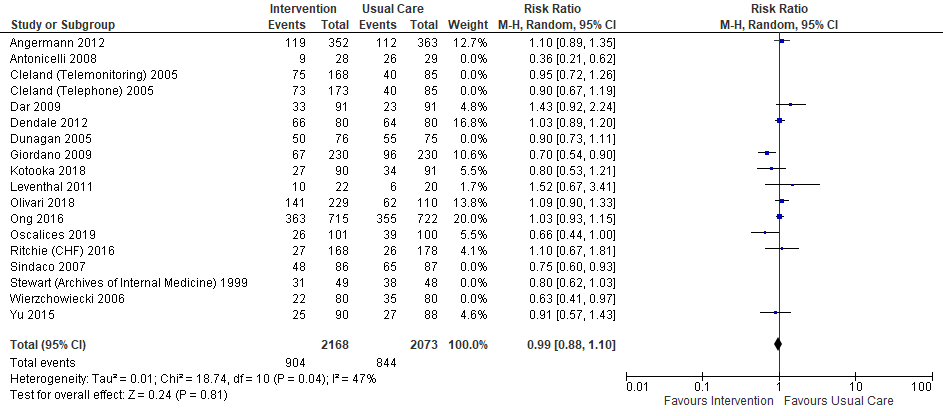

Supplement: S4 Fig — (DOCX) [file pone.0249542.s005.docx]

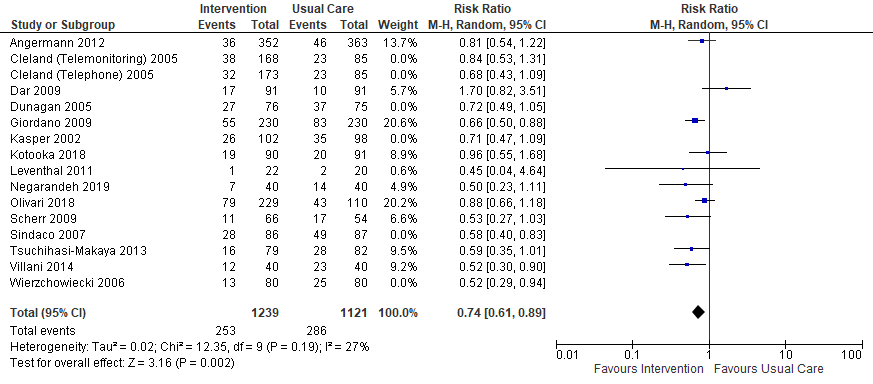

Supplement: S5 Fig — (DOCX) [file pone.0249542.s006.docx]

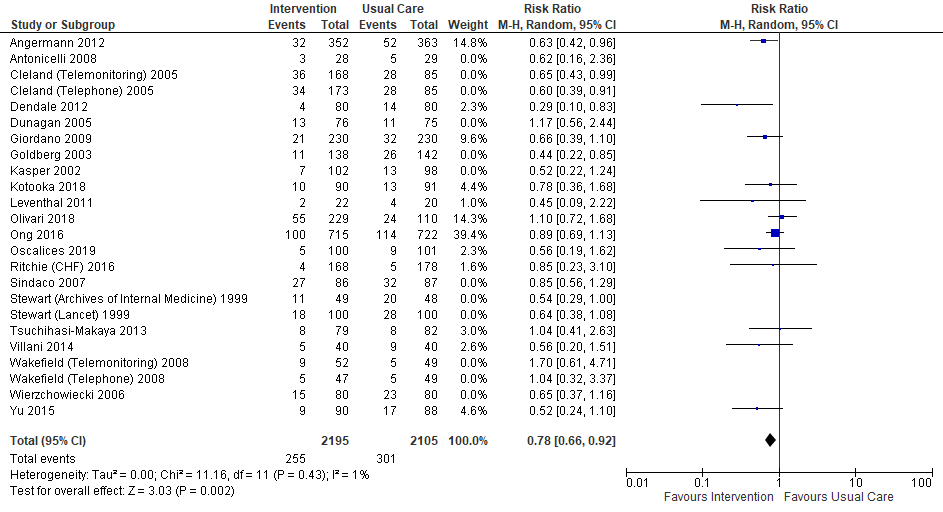

Supplement: S6 Fig — (DOCX) [file pone.0249542.s007.docx]

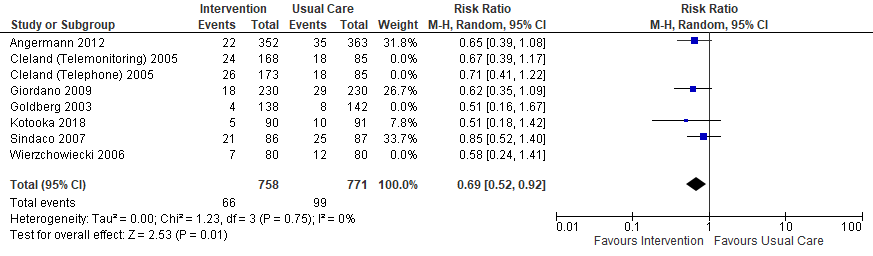

Supplement: S7 Fig — (DOCX) [file pone.0249542.s008.docx]
